# Resampling Methods Improve the Predictive Power of Modeling in Class-Imbalanced Datasets

**DOI:** 10.3390/ijerph110909776

**Published:** 2014-09-18

**Authors:** Paul H. Lee

**Affiliations:** School of Nursing, Hong Kong Polytechnic University, Hung Hom, Kowloon, Hong Kong; E-Mail: paul.h.lee@polyu.edu.hk; Tel.: +852-3400-8275; Fax: +852-2364-9663

**Keywords:** automated classifier, data mining, decision tree, oversampling, predictive power, rare events

## Abstract

In the medical field, many outcome variables are dichotomized, and the two possible values of a dichotomized variable are referred to as classes. A dichotomized dataset is class-imbalanced if it consists mostly of one class, and performance of common classification models on this type of dataset tends to be suboptimal. To tackle such a problem, resampling methods, including oversampling and undersampling can be used. This paper aims at illustrating the effect of resampling methods using the National Health and Nutrition Examination Survey (NHANES) wave 2009–2010 dataset. A total of 4677 participants aged ≥20 without self-reported diabetes and with valid blood test results were analyzed. The Classification and Regression Tree (CART) procedure was used to build a classification model on undiagnosed diabetes. A participant demonstrated evidence of diabetes according to WHO diabetes criteria. Exposure variables included demographics and socio-economic status. CART models were fitted using a randomly selected 70% of the data (training dataset), and area under the receiver operating characteristic curve (AUC) was computed using the remaining 30% of the sample for evaluation (testing dataset). CART models were fitted using the training dataset, the oversampled training dataset, the weighted training dataset, and the undersampled training dataset. In addition, resampling case-to-control ratio of 1:1, 1:2, and 1:4 were examined. Resampling methods on the performance of other extensions of CART (random forests and generalized boosted trees) were also examined. CARTs fitted on the oversampled (AUC = 0.70) and undersampled training data (AUC = 0.74) yielded a better classification power than that on the training data (AUC = 0.65). Resampling could also improve the classification power of random forests and generalized boosted trees. To conclude, applying resampling methods in a class-imbalanced dataset improved the classification power of CART, random forests, and generalized boosted trees.

## 1. Introduction

In the medical field, many outcome variables are dichotomized (or binary), for example survival status, indicator of a particular disease, and adequacy of a particular nutrient. The two possible values of a dichotomized variable are referred to as classes. A dataset with dichotomized outcome is class-imbalanced if it consists mostly of one class. Class-imbalance is a common phenomenon in the medical context; analysis of rare diseases or mortality within a short period of follow-up usually suffers from class-imbalance problems. Class-imbalanced datasets are challenging to analyze, as the performance of common classification models (for example decision trees) on class-imbalanced datasets tends to be suboptimal [1] since these models target to improve the overall accuracy, hence these models focus on classifying correctly the large class at the cost of ignoring the misclassification of the small class. However, the actual cost of misclassifying the small class (false negative) maybe much higher than misclassification of the large class (false positive).

To tackle such a problem arising from class-imbalance, a classic solution is to use a case-control study design [2,3]. In adopting a case-control study design, researchers first draw samples of the cases (supposing that the case is a rare event), then samples of the controls are drawn according to the collected samples of the cases. The advantage of a case-control study over a cohort study is that the case-control study does not require a large sample size and long follow-up period to accumulate a reasonable number of rare disease patients.

However, a dataset obtained using case-control study design is only suitable for estimating the relative risk or odds ratio of several exposures on the particular disease. If the study objective is to estimate the risk factor of more than one disease, cohort or cross-sectional study designs appear to be more appropriate. In studies using cohort and cross-sectional designs, problem arising from class-imbalance can be tackled at the stage of statistical analysis using resampling methods [4]. Resampling methods include oversampling, *i.e*., oversample the small class to a sample size comparable to the large class, and undersampling, *i.e*., randomly draw samples from the large class with sample size comparable to the small class. A lot of work had been done in the data mining literature on developing resampling methods [5], yet these techniques are rarely applied in the medical literature.

This paper aims at illustrating the effect of resampling methods in medical research, using the public-available National Health and Nutrition Examination Survey (NHANES) wave 2009–2010 data. Using these data, we built several decision tree models to predict undiagnosed diabetes among adult participants. According to the Centers for Disease Control and Prevention, the prevalence of diagnosed and undiagnosed diabetes are 6.0% and 2.3%, respectively [6], and given its large burden to society [7], a huge effort was dedicated to identify undiagnosed diabetes for better decision-making of health care providers. A recent systematic review showed that from 1997 to 2010 there were 15 published papers about developing prediction models to identify undiagnosed diabetes [8], but none of these addressed the problem of class imbalance. In the NHANES 2009–2010 data, the prevalence of undiagnosed diabetes among adults age ≥20 without self-report diabetes was 3.0% (shown below). Here, using the NHANES 2009–2010 data, we compare the predictive power of the decision tree models on the full dataset and on the resampled datasets and it was hypothesized that using the resampled dataset the predictive power will be improved. Here we also consider unbalanced resampling, that is, resampling to a pre-determined ratio of both classes. Resampling to different rates had been studied and these sometimes yield better predictive power than balanced resampling [4].

## 2. Methods

### 2.1. Ethics Statement

The NHANES study was approved by the Centers for Disease Control and Prevention ethics review board (Continuation of Protocol #2005-06). The NHANES also obtained consent from all participants.

### 2.2. Participants

This study utilized data collected from participants in the National Health and Nutrition Examination Survey (NHANES) wave 2009–2010. The NHANES, conducted by the National Center for Health Statistics, Centers for Disease Control and Prevention, was designed to assess the health and nutrition status in the United States [9]. The sample was representative of the United States population, and was selected using a multi-stage probability cluster design. Participants were invited to complete a survey and a health examination; the details can be obtained from the NHANES website [10]. A total of 10,537 participants completed the survey, and those with aged 19 and below, with self-reported diabetes (that is, having a positive response in the question “Have you ever been told by a doctor or health professional that you have diabetes or sugar diabetes?”), and/or without blood test results were excluded in this study, leaving a final sample of 4677.

### 2.3. Measurement

A blood test was conducted in a morning examination after a 9-hour fast to obtain fasting glucose and hemoglobin A1c levels of the participants. In addition, a two-hour oral glucose tolerance test was conducted to obtain non-fasting glucose level. A participant demonstrated evidence of diabetes if any of the following is met: (a) fasting glucose ≥ 126 mg/dL, (b) non-fasting glucose ≥ 200 mg/dL, (c) hemoglobin A1c ≥ 6.5% (or 47.5 mmol/mol). BMI was calculated as weight (kg) divided by the square of height (m^2^). Family Poverty Index, determined by the eligibility of certain federal financial assistance programs, was computed according to the Department of Health and Human Services guidelines. The Family Poverty Income Ratio was computed by dividing the Family income by the Family Poverty Index. Other exposure variables included age, race, marital status, and education level. To facilitate the use of decision tree models in non-clinical setting, relevant biomarkers, e.g., blood pressure or high-density lipoprotein, were not included as exposure variables.

### 2.4. Statistical Analysis

**Figure 1:**
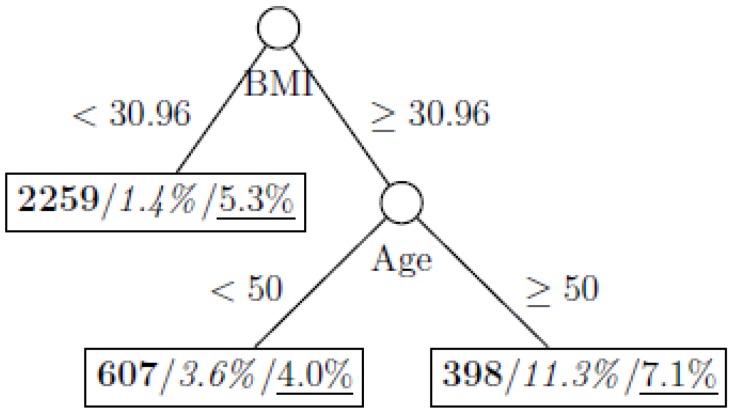
Decision tree model fitted using the full training dataset (number in **bold**/*italic*/underline: sample size of the node/percentage of undiagnosed diabetes in the training dataset/percentage of undiagnosed diabetes in the testing dataset respectively).

**Figure 2:**
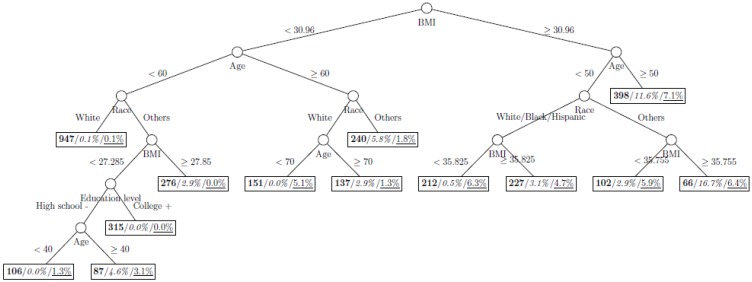
Decision tree model fitted using the weighted training dataset (number in **bold**/*italic*/underline: sample size of the node/percentage of undiagnosed diabetes in the training dataset/percentage of undiagnosed diabetes in the testing dataset respectively).

**Figure 3:**
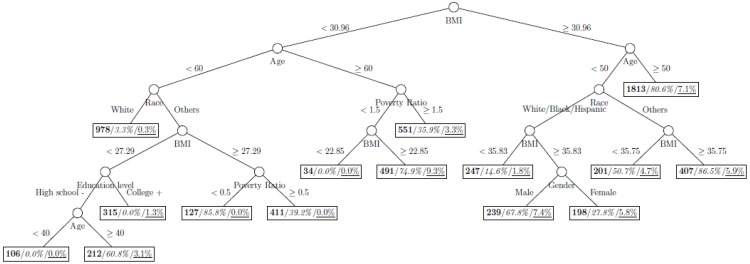
Decision tree model fitted using the oversampled (case-to-control ratio = 1:1) training dataset (number in **bold**/*italic*/underline: sample size of the node/percentage of undiagnosed diabetes in the training dataset/percentage of undiagnosed diabetes in the testing dataset respectively).

**Figure 4:**
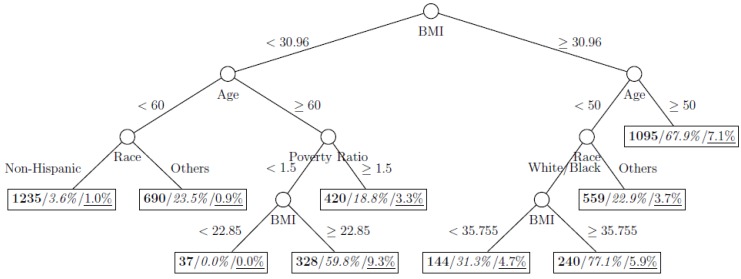
Decision tree model fitted using the oversampled (case-to-control ratio = 1:2) training dataset (number in **bold**/*italic*/underline: sample size of the node/percentage of undiagnosed diabetes in the training dataset/percentage of undiagnosed diabetes in the testing dataset respectively).

**Figure 5:**
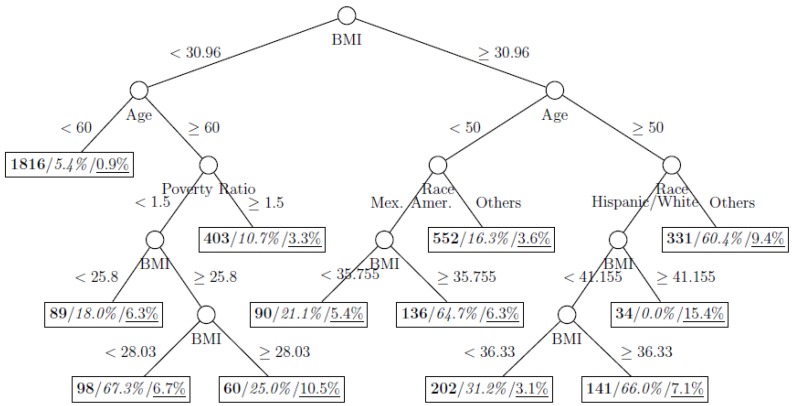
Decision tree model fitted using the oversampled (case-to-control ratio = 1:4) training dataset (number in **bold**/*italic*/underline: sample size of the node/percentage of undiagnosed diabetes in the training dataset/percentage of undiagnosed diabetes in the testing dataset respectively).

**Figure 6:**
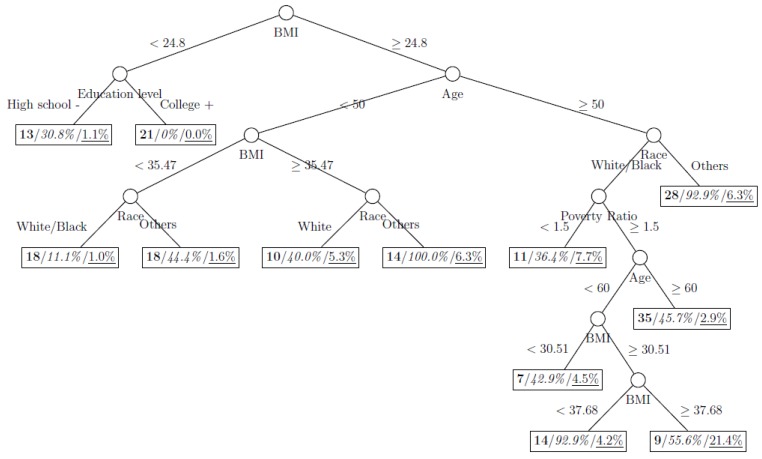
Decision tree model fitted using the undersampled (case-to-control ratio = 1:1) training dataset (number in **bold**/*italic*/underline: sample size of the node/percentage of undiagnosed diabetes in the training dataset/percentage of undiagnosed diabetes in the testing dataset respectively).

**Figure 7:**
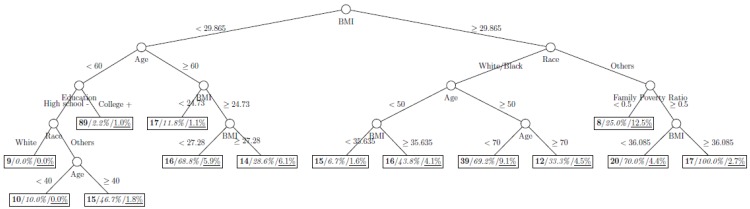
Decision tree model fitted using the undersampled (case-to-control ratio = 1:2) training dataset (number in **bold**/*italic*/underline: sample size of the node/percentage of undiagnosed diabetes in the training dataset/percentage of undiagnosed diabetes in the testing dataset respectively).

**Figure 8:**
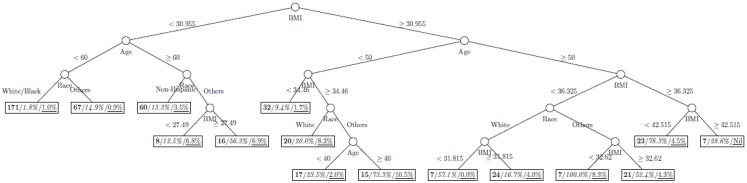
Decision tree model fitted using the undersampled (case-to-control ratio = 1:4) training dataset (number in **bold**/*italic*/underline: sample size of the node/percentage of undiagnosed diabetes in the training dataset/percentage of undiagnosed diabetes in the testing dataset respectively).

The Classification and Regression Tree (CART) procedure [11] was used to build a classification model on undiagnosed diabetes. CART is a recursive partitioning procedure aim at splitting the data into distinct partitions base on the most important exposure variables determined by the procedure. A split on a partition is carried out to maximize the purity, that is, the dominance of one class, of its descendant partitions. In this study, the purity of a partition is measured by the Gini impurity, which equals 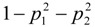 where *p*_1_ and *p*_2_ are the proportions of classes 1 and 2 respectively. The model is named as a tree model as the partitions can be arranged in a tree-like structure, as shown in Figure 1, Figure 2, Figure 3, Figure 4, Figure 5, Figure 6, Figure 7 and Figure 8. The CART was fitted using package *rpart* of R, with a complexity parameter and minimum number of partition size of 0.01 and 20, respectively. We also fitted CART model with complexity parameter determined by the 1-SE rule (a standard, accepted method for complexity parameter determination) [9], the random forest model using package *randomForest* of R with 500 trees, and the generalized boosted trees using package *gbm* of R with 100 trees. Random forests and generalized boosted trees are extension of CART models by constructing multiple decision trees to improve prediction accuracy.

Decision tree models were built with demographic characteristics as predictors including age, sex, education, race, BMI, and Family Poverty Ratio. Demographic characteristics were found predictive for undiagnosed diabetes [8]. To assess the classification power of the decision tree models, the models are fitted using a randomly selected 70% of the data (*n* = 3264, named as training dataset), and the area under the receiver operating characteristic curve (AUC), sensitivity, specificity, positive predictive value (PPV), negative predictive value (NPV), and classification rate were computed using the remaining 30% (*n* = 1413, 40 of them had undiagnosed diabetes) of the sample for evaluation (named as testing dataset). AUC is the most commonly used indicator for model comparison [12] and a value of 0.70 or above was considered as good fit [13].

Among the 3264 participants in the training dataset, 3165 did not have any evidences of diabetes and the remaining 99 had diabetes. The CART was fitted using eight datasets: (a1) the training dataset (*n* = 3264), (a2) the weighted training dataset with diabetes to non-diabetes participants ratio of 1:1 (*n* = 3264), (b1) the oversampled dataset sample that combined randomly oversampled 3165 diabetes participants from the original 99 diabetes participants with the 3165 participants without diabetes (*n* = 6330), (b2) the oversampled training dataset with case-to-control ratio of 1:2 (*n* = 4,748), (b3) the oversampled training dataset with case-to-control ratio of 1:4 (*n* = 3957), (c1) the undersampled training dataset that combined randomly selected 99 participants without diabetes out of the 3165 with the 99 diabetes participants (*n* = 198), (c2) the undersampled training dataset with case-to-control ratio of 1:2 (*n* = 297), and (c3) the undersampled training dataset with case-to-control ratio of 1:4 (*n* = 495). Method of modifying the loss matrix of CART that adjusts the weightings on false positive rate and false negative rate was not adopted here as it had no effect on the tree built.

## 3. Results

Table 1 shows the descriptive statistics of the training and testing datasets. There were no differences between training and testing datasets for all exposure variables (all *p* > 0.05). There was no difference (χ^2^ = 0.14, *p* = 0.71) in the incidence rates of diabetes in the training dataset (*n* = 99, 3.0%) and the testing dataset (*n* = 40, 2.8%). Among participants in the training dataset, those with undiagnosed diabetes consisted of more of aged 50 or above and Mexican American, had less than nine years of education, had lower Family Poverty Income Ratio, and had higher BMI.

**Table 1:** Characteristics of the training and testing datasets.

Variable (categorical)	Training dataset (*n* = 3264)		Testing dataset (*n* = 1413)	Total (*n* = 4677)
	No diabetes (*n* = 3165)	Diabetes (*n* = 99)		
	Frequency (%)	Frequency (%)	Frequency (%)	Frequency (%)
Gender				
Male	1523 (48.1%)	47 (47.5%)	669 (47.3%)	2239 (47.9%)
Female	1642 (51.9%)	52 (52.5%)	744 (52.7%)	2438 (52.1%)
Age ***				
20–29	656 (20.7%)	5 (5.1%)	300 (21.2%)	961 (20.5%)
30–39	631 (19.9%)	9 (9.1%)	273 (19.3%)	913 (19.5%)
40–49	650 (20.5%)	16 (16.2%)	285 (20.2%)	951 (20.3%)
50–59	515 (16.3%)	19 (19.2%)	201 (14.2%)	735 (15.7%)
60–69	431 (13.6%)	35 (35.4%)	195 (13.8%)	661 (14.1%)
70 or above	282 (8.9%)	15 (15.2%)	159 (11.3%)	456 (9.7%)
Race ***				
Mexican American	557 (17.6%)	34 (34.3%)	287 (20.3%)	878 (18.8%)
Other Hispanic	346 (10.9%)	11 (11.1%)	129 (9.1%)	486 (10.4%)
Non-Hispanic White	1549 (48.9%)	27 (27.3%)	674 (47.7%)	2250 (48.1%)
Non-Hispanic Black	527 (16.7%)	22 (22.2%)	256 (18.1%)	805 (17.2%)
Other race including multi-racial	186 (5.9%)	5 (5.1%)	67 (4.7%)	258 (5.5%)
Education level *				
Less than 9th grade	331 (10.5%)	17 (17.2%)	169 (12.0%)	517 (11.1%)
9th–11th grade	506 (16.0%)	21 (21.2%)	209 (14.8%)	736 (15.8%)
High school	743 (23.5%)	27 (27.3%)	321 (22.8%)	1091 (23.4%)
Some college	890 (28.2%)	24 (24.2%)	406 (28.8%)	1320 (28.3%)
College graduate or above	689 (21.8%)	10 (10.1%)	305 (21.6%)	1004 (21.5%)
Missing	6	0	3	9
Marital status				
Married	1620 (51.2%)	52 (52.5%)	751 (53.2%)	2423 (51.8%)
Widowed	151 (4.8%)	9 (9.1%)	76 (5.4%)	236 (5.0%)
Divorced	368 (11.6%)	9 (9.1%)	125 (8.9%)	502 (10.7%)
Separated	106 (3.4%)	6 (6.1%)	53 (3.8%)	165 (3.5%)
Never married	621 (19.6%)	12 (12.1%)	281 (19.9%)	914 (19.6%)
Living with partner	298 (9.4%)	11 (11.1%)	126 (8.9%)	435 (9.3%)
Missing	1	0	1	2
**Variable (continuous)**	Mean (SD)	Mean (SD)	Mean (SD)	Mean (SD)
Family Poverty Income Ratio *	2.46 (1.66)	2.07 (1.50)	2.47 (1.63)	2.46 (1.65)
Body Mass Index ***	28.67 (6.61)	33.74 (6.50)	28.65 (6.68)	28.77 (6.67)

*/**/*** χ^2^ test between no diabetes and diabetes in training dataset significant at 5%/1%/0.1% level. All χ^2^ tests between training dataset and testing dataset were insignificant at 5% level.

Figure 1, Figure 2, Figure 3, Figure 4, Figure 5, Figure 6, Figure 7 and Figure 8 show the decision tree model fitted using the CART algorithm on the full, weighted, oversampled (case-to-control ratio 1:1, 1:2, and 1:4), and undersampled (case-to-control ratio 1:1, 1:2, and 1:4) training dataset respectively. They have 3, 13, 12, 14, 15, 15, 9, and 12 partitions respectively. Tree on full training data included two (BMI and age) exposure variables and other trees included four to six exposure variables. It was obvious that the tree model fitted on the full training dataset was an underfit. In fact, the tree model fitted on the oversampled training dataset was an extension of that on the full training dataset, with the partitions “BMI < 30.96” and “BMI ≥ 30.96 and Age < 50” further split into nine and five partitions respectively. The decision trees across different case-to-control ratios were similar. In decision trees fitted on the oversampled and undersampled training dataset with case-to-control ratio of 1:1, the partition having the highest incidence of diabetes had similar characteristics (oversampled: BMI ≥ 35.47 and Age < 50 and Race = others, undersampled: 30.96 > BMI ≥ 27.29 and Age < 60 and Race = other and Family Poverty Income Ratio < 0.5). The decision trees fitted on the weighted training dataset was nearly the same with that on the oversampled training dataset with case-to-control ratio of 1:1.

**Figure 9:**
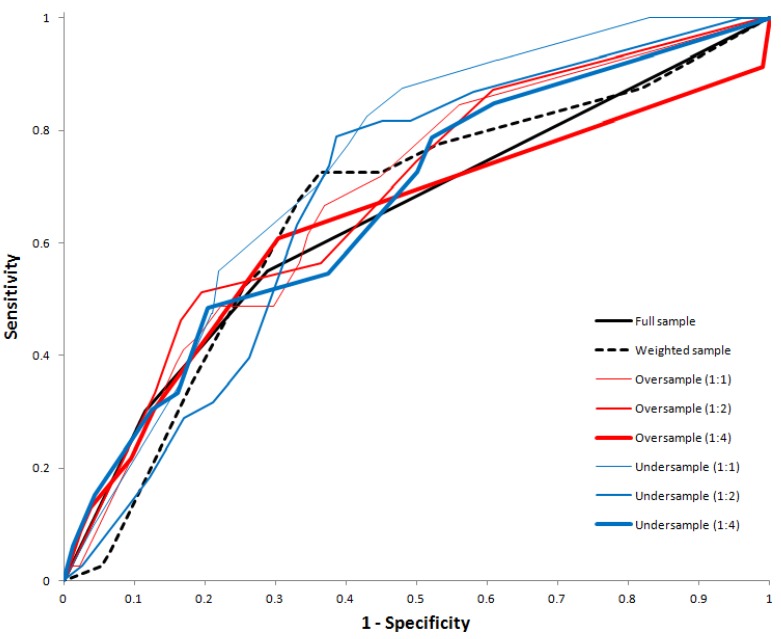
Receiver operating characteristic (ROC) curve for the decision tree models fitting using the full, weighted, oversampled, and undersampled training dataset.

Table 2 shows the classification performance of all decision trees. While NPV were similar across all decision trees, the classification rate of trees on the full training data and the weighted datasets were substantially smaller than those with resampled training datasets. Both the trees fitted on the oversampled and undersampled training data with case-to-control ratio of 1:1 yielded a good fit with AUC above 0.70, however the tree on the full training data, the weighted training data and the resampled training data with case-to-control ratios of 1:2 and 1:4 did not yield a good fit with an AUC of 0.63 to 0.69. There is a clear trend that the AUC reduce with case-to-control ratio for both oversampled and undersampled training dataset. Figure 9 shows the receiver operating characteristic curves of all tree models.

**Table 2:** Classification power of the Classification and Regression Tree (CART) Models in the testing dataset.

Performance indicator	Full dataset	Weighted dataset	Oversampled dataset			Undersampled dataset		
			Case-to-control ratio			Case-to-control ratio		
			1:1	1:2	1:4	1:1	1:2	1:4
AUC	0.65	0.65	0.70	0.69	0.63	0.74	0.68	0.67
Sensitivity	55.0%	67.5%	48.7%	46.2%	30.4%	55.0%	63.2%	48.5%
Specificity	71.1%	66.7%	77.7%	83.4%	87.2%	78.0%	67.0%	79.6%
PPV	5.3%	5.6%	6.0%	7.5%	4.8%	6.8%	5.1%	5.6%
NPV	98.2%	98.6%	98.1%	98.2%	98.3%	98.3%	98.5%	98.4%
Classification rate	70.6%	66.7%	76.9%	82.4%	86.0%	77.4%	66.9%	78.8%

AUC: area under the receiver operating characteristic curve; PPV: positive predictive value; NPV: negative predictive value.

The classification performance of the decision trees with complexity parameter determined by 1-SE rule, the random forest models, and the generalized boosted trees, can be found in Table 3, Table 4 and Table 5 respectively. The decision tree model fitted on the full training dataset showed poor AUC as it had no splitting at all. Only the tree on the weighted training dataset and the undersampled training datasets demonstrated good fit. Not limited in CART models, resampling methods could also improve classification power for random forest models and generalized boosted trees. For random forest models, only those fitted on undersampled training datasets demonstrated good fit (Table 4). For generalized boosted trees, those fitted on full training dataset, weighted training dataset, and undersampled training datasets demonstrated good fit, and the undersampled dataset with case-to-control ratio 1:1 yielded the best classification power, although the specificity is zero (Table 5).

By comparing the classification performance of different types of models (Table 2, Table 3, Table 4 and Table 5), we can see that undersampling could improve the classification power of all models. Most of the models fitted using the undersampled dataset could achieve an AUC of above 0.7 and in general the case-to-control ratio of 1:1 performed the best.

**Table 3:** Classification power of the Classification and Regression Tree (CART) Models (complexity parameter determined by 1-SE rule) in the testing dataset.

Performance indicator	Full dataset	Weighted dataset	Oversampled dataset			Undersampled dataset		
			Case-to-control ratio			Case-to-control ratio		
			1:1	1:2	1:4	1:1	1:2	1:4
AUC	0.50	0.71	0.54	0.58	0.61	0.73	0.71	0.73
Sensitivity	0%	15.0%	15.0%	22.5%	25.0%	67.5%	55.0%	62.5%
Specificity	100%	92.1%	92.1%	92.3%	88.5%	73.0%	75.2%	74.2%
PPV	N/A	5.3%	5.3%	7.8%	6.0%	6.8%	6.1%	6.6%
NPV	97.2%	97.4%	97.4%	97.6%	97.6%	98.7%	98.3%	98.5%
Classification rate	97.2%	90.0%	90.0%	90.3%	86.7%	72.8%	74.7%	73.9%

AUC: area under the receiver operating characteristic curve; PPV: positive predictive value; NPV: negative predictive value.

**Table 4:** Classification power of the random forest models in the testing dataset.

Performance indicator	Full dataset	Weighted dataset	Oversampled dataset			Undersampled dataset		
			Case-to-control ratio			Case-to-control ratio		
			1:1	1:2	1:4	1:1	1:2	1:4
AUC	0.68	0.69	0.69	0.68	0.68	0.76	0.76	0.75
Sensitivity	54.3%	51.4%	11.4%	14.3%	22.9%	82.9%	68.6%	71.4%
Specificity	75.1%	75.2%	97.6%	96.0%	90.7%	59.6%	70.5%	70.0%
PPV	5.7%	5.5%	11.8%	9.1%	6.4%	5.4%	6.1%	6.2%
NPV	98.3%	98.2%	97.5%	97.6%	97.7%	99.2%	98.8%	98.9%
Classification rate	74.5%	74.5%	95.3%	93.8%	88.8%	60.2%	70.4%	70.0%

AUC: area under the receiver operating characteristic curve; PPV: positive predictive value; NPV: negative predictive value.

**Table 5:** Classification power of the generalized boosted tree models in the testing dataset.

Performance indicator	Full dataset	Weighted dataset	Oversampled dataset			Undersampled dataset		
			Case-to-control ratio			Case-to-control ratio		
			1:1	1:2	1:4	1:1	1:2	1:4
AUC	0.74	0.73	0.64	0.68	0.65	0.79	0.72	0.72
Sensitivity	55.0%	55.0%	55.0%	55.0%	55.0%	100%	60.0%	52.5%
Specificity	70.6%	70.6%	70.6%	70.6%	70.6%	0%	66.1%	71.9%
PPV	5.2%	5.2%	5.2%	5.2%	5.2%	2.8%	4.9%	5.2%
NPV	98.2%	98.2%	98.2%	98.2%	98.2%	N/A	98.3%	98.1%
Classification rate	70.2%	70.2%	70.2%	70.2%	70.2%	2.8%	66.0%	71.3%

AUC: area under the receiver operating characteristic curve; PPV: positive predictive value; NPV: negative predictive value.

## 4. Discussion

Illustrated with a dataset with only 3.0% of the participants classified as undiagnosed diabetes, our results showed that applying resampling methods in a class-imbalanced dataset clearly improved the explanatory power of the decision tree models, random forests, and generalized boosted trees. With an illustration from a public health perspective, a systematic comparison between standard method of analysis and those based on resampled data showed that resampling could improve the overall classification rate and positive predictive value. Besides CART, the performance of other extended tree models including random forests and generalized boosted trees could also be improved using resampling. The decision tree fitted on the full training dataset clearly underfitted the data and this could be explained as follows. By comparing the trees on the full training dataset and the overersampled dataset with case-to-control ratio of 1:1, we can see that the split for the partition “BMI < 30.96” stopped in the former model but continued to split in the latter model. It is because the reduction of Gini impurity for the split “Age < 60 *vs*. Age 60” is minimal (from 1 – (31/2259)^2^ − (2228/2259)^2^ = 0.0271 to (1731/2259) × (1 – (13/1731)^2^ − (1718/1731)^2^) + (528/2259) × (1 – (18/528)^2^ − (510/528)^2^ = 0.0268), while using the oversampled training data this reduction can be amplified (from 1 – (997/3225)^2^ − (2228/3225)^2^ = 0.4272 to (2149/3225) × (1 – (431/2149)^2^ − (1718/2149)^2^) + (1076/3225) × (1 – (566/1076)^2^ − (510/1076)^2^ = 0.3801).

As the use of automated classifiers like decision trees, support vector machines (see [14] for example) and artificial neural networks (see [15] for example) are becoming much more popular in the medical literature, the use of resampling methods should be promoted as it apply on all these statistical models that targeting at maximizing accuracy [16]. (Re)Analysis of previously published data using resampling methods is warranted given the potential suboptimal results of existing analyses.

The most commonly applied statistical model for predicting undiagnosed diabetes was the logistic regression [8]. Although there was no evidence that resampling methods improve the predictive power of logistic regression or even any class of generalized linear models, applying logistic regression on a class-imbalanced dataset may sometimes be inappropriate, especially when there are a large number of exposure variables. Generalized linear models require as much as 10 to 20 cases of both classes per exposure variable [17,18], and if such models were applied on our example, only 5 to 10 exposures variables were allowed. This is obviously too strict a criterion for predictive models for undiagnosed diabetes given its multi-factorial nature [8]. Therefore, given such an imbalanced dataset, modeling using automated classifiers appears to be the only appropriate choice and the dataset should be resampled.

In this study, we consider both balanced and unbalanced resampling. However, resampling methods combining both oversampled and undersampled datasets [4], have not been examined. Besides these extensions on resampling methods, researchers had also developed non-random resampling methods to reduce the potential bias and overfitting caused by random resampling [19,20]. However, these advanced resampling methods had been rarely applied in the medical literature. Given the effectiveness of the balanced resampling methods that have been shown in this study, the effectiveness of these advanced resampling methods is worth exploring.

Our study was not without limitations. First, biomarkers that were found associated with undiagnosed diabetes, for example blood pressure, high-density lipoprotein, C-reactive protein, triaglyceride, and white blood cell count [8] were not included in the decision tree model for facilitating the use of decision tree models in non-clinical setting. Improvement of resampling methods on models including these biomarkers was unknown; however we believe that resampling should be effective as well. Second, only decision tree models and their extensions, but not other automatic classifiers, were employed, as decision tree models, but not other automatic classifiers, are feasible to be administered in a clinical setting to predict undiagnosed diabetes patients, albeit their underperformance compared with other classifiers such as ensemble methods [21]. Further research on the effectiveness of resampling methods with support vector machines and artificial neural networks is warranted. Note that oversampling will introduce dependence to the data, therefore using traditional regression models, which assume data independence, on oversampled data may not be appropriate.

## 5. Conclusions

Our results showed that applying resampling methods in a class-imbalanced dataset clearly improved the classification power of the CART model, random forests, and generalized boosted tree. Data analysis targeting at maximizing accuracy should apply resampling methods.
